# The *ALLgorithMM*: How to define the hemodilution of bone marrow samples in lymphoproliferative diseases

**DOI:** 10.3389/fonc.2022.1001048

**Published:** 2022-10-06

**Authors:** Ilaria Vigliotta, Silvia Armuzzi, Martina Barone, Vincenza Solli, Ignazia Pistis, Enrica Borsi, Barbara Taurisano, Gaia Mazzocchetti, Marina Martello, Andrea Poletti, Chiara Sartor, Ilaria Rizzello, Lucia Pantani, Paola Tacchetti, Cristina Papayannidis, Katia Mancuso, Serena Rocchi, Elena Zamagni, Antonio Curti, Mario Arpinati, Michele Cavo, Carolina Terragna

**Affiliations:** ^1^ IRCCS Azienda Ospedaliero-Universitaria di Bologna, Seràgnoli Institute of Hematology, Bologna, Italy; ^2^ Department of Experimental, Diagnostic and Specialty Medicine - University of Bologna, Bologna, Italy

**Keywords:** minimal residual disease, multiple myeloma, acute lymphoblastic leukemia, hemodilution, hemodilution/methods, flow cytometry, measurable (minimal) residual disease

## Abstract

**Introduction:**

Minimal residual disease (MRD) is commonly assessed in bone marrow (BM) aspirate. However, sample quality can impair the MRD measurement, leading to underestimated residual cells and to false negative results. To define a reliable and reproducible method for the assessment of BM hemodilution, several flow cytometry (FC) strategies for hemodilution evaluation have been compared.

**Methods:**

For each BM sample, cells populations with a well-known distribution in BM and peripheral blood - e.g., mast cells (MC), immature (IG) and mature granulocytes (N) – have been studied by FC and quantified alongside the BM differential count.

**Results:**

The frequencies of cells’ populations were correlated to the IG/N ratio, highlighting a mild correlation with MCs and erythroblasts (R=0.25 and R=0.38 respectively, with p-value=0.0006 and 0.0000052), whereas no significant correlation was found with B or T-cells. The mild correlation between IG/N, erythroblasts and MCs supported the combined use of these parameters to evaluate BM hemodilution, hence the optimization of the *ALLgorithMM*. Once validated, the *ALLgorithMM* was employed to evaluate the dilution status of BM samples in the context of MRD assessment. Overall, we found that 32% of FC and 52% of Next Generation Sequencing (NGS) analyses were MRD negative in samples resulted hemodiluted (HD) or at least mildly hemodiluted (mHD).

**Conclusions:**

The high frequency of MRD-negative results in both HD and mHD samples implies the presence of possible false negative MRD measurements, impairing the correct assessment of patients’ response to therapy and highlighs the importance to evaluate BM hemodilution.

## Introduction

The study of minimal residual disease (MRD) provides critical information for the management of hematological patients affected by lymphoproliferative diseases, representing the best biomarker to monitor treatments’ efficacy and to define the eradication of residual tumor cells. Moreover, MRD is increasingly taking hold for the choice of therapy, whether for the modulation of therapeutic intensity, for the indication of stem cell transplantation or eventually for treatment discontinuation (1–7). International guidelines are recommending the use of bone marrow (BM) aspirate, with specific sensitivity thresholds, as gold standard for MRD measurements in most hematological malignancies, in particular Multiple Myeloma (MM) (1, 2, 8–10) and Acute Lymphoblastic Leukemia (ALL) (4, 11–13).

However, one of the main pitfalls of the MRD quantification is represented by the quality of the sample itself, as the dilution of the tumor cells with peripheral blood (PB) (defined as hemodilution) that occurs within BM sampling can cause an underestimation of residual disease cells, and lead to biased MRD evaluation. Considering the growing role of MRD evaluation both for prognosis and for treatment tailoring (14, 15), it seems more and more necessary to investigate whether the cases of negativity would not actually be linked to bad sampling of the BM specimen, particularly in those settings involving T cell redirecting therapies (e.g., engineered chimeric antigen receptor (CAR) T-cells).

To date, no consensus has been established yet, both on criteria for samples’ quality acceptability and on the set up of standardized protocols for hemodilution evaluation. In fact, over time, several parameters have been investigated to define hemodilution in different diseases by distinct groups, such as the Holdrinet index (16), the frequencies of plasma cells and CD34+ cells (17), the ratio between immature and mature granulocytes (18), mast cells and hematogones presence (19) among others.

Thus, the aim of the current study was to define a robust and reproducible method for the assessment of BM hemodilution, applicable to lymphoproliferative diseases, in order to ensure the best quality of MRD measurements and interpretation.

## Materials and methods

### Patient cohort

All patients included in the study provided written informed consents for biological studies and have been treated following local recommendations for clinical trials or routine clinical practice at the Seràgnoli Institute, IRCCS Azienda Ospedaliero-Universitaria of Bologna, Italy. An initial cohort of 78 Multiple Myeloma (MM) and 18 Acute Lymphoblastic Leukemia (ALL) patients (104 and 34 samples, respectively) was used to set-up the experimental plan to evaluate the cellular immunophenotype by flow cytometry (FC) of the most representative BM cell types and to create the matrix to compare diverse hemodilution strategies, previously described by other groups (16–20). ALL patients are represented by Philadelphia (Ph)-negative B-ALL or T-ALL patients, for whom minimal residual disease (MRD) is measured molecularly by Next Generation Sequencing (NGS). NGS was employed for MRD monitoring in MM patients, as well. All patients and cohort characteristics are shown in **Table 1**. Briefly, patients had a median age of 46 (range 20-77) and 61 (range 39-76) years old for ALL and MM, respectively; as for the gender, 65% (39/60) of ALL patients and 56% (74/133) of MM were male. ALL patients were mostly treated with chemotherapy by pediatric-like regimens and/or by reduced regimens in elderly patients (10/25; 40%); 8/25 patients (32%) were relapsed/refractory or MRD-positive under Inotuzumab ozogamicin and/or Blinatumobab at the time of BM sampling, whereas in 6/25 patients (24%) MRD was evaluated after BM transplant. One patient was not under treatment, when BM was collected. Most MM patients (82/91; 90%) were transplant-eligible: in most cases (84%) MRD samples were collected during maintenance, whereas in the remaining (16%) post-induction. The majority (54%) of MM patients were under Immunomodulatory drugs (IMiDs) regimens at the time of BM sampling, whereas others were under treatment with anti-CD38 (15%) or Proteasome Inhibitors (PI; 23%) in the context of outpatient regimen and/or within clinical trials.

**Table 1:** Patients’ overview. Three main sub-groups of patients have been included in the study: Acute Lymphoblastic Leukemia (ALL) patients, Multiple Myeloma (MM) patients and MM patients treated with chimeric antigen receptor (CAR)- T cells.

	Patients	Samples	Age (range)	Gender
**ALL**	25	69	46 (20-77)	F^2^ 35%-M 65%
**MM**	91	150	61 (39-76)	F 44%-M 56%
**CAR-T^1^**	9	14	54 (38-64)	F 36%-M 64%
**Tot**	125	233	54 (20-77)	F 38%-M 62%

^1^CAR-T is referred only to MM patients who were sampled after at least 56 days after anti-BCMA CAR-T infusion (median: 171 days; range: 56-365 days); ^2^F, female and M, male.

In the validation phase of the study, CAR-T-treated MM patients (9/100; 9%) (i.e., anti-B-cell maturation antigen (BCMA) CAR-T) have been added to the cohort. In these cases, since CAR-T cells therapy, preceded by lymphodepletion chemotherapy, could cause a modulation of BM cell populations (19), we decided not to perform hemodilution evaluations on BM samples taken from patients who had undergone the CAR-T infusion within 56 days (median: 171 days; range: 56-365 days).

For each patient, at least 4 ml of BM aspirates were collected, deriving from a single-site iliac crest sampling and samples were preserved in EDTA tubes for subsequent analyses (e.g. MRD evaluation).

### Flow cytometry strategy

Cell distribution within BM aspirates, in terms of quantification and characterization, was assessed FC and all analyses were performed within 24 hours from BM sampling. Cellular immunophenotype was analyzed *via* FACSCanto™ II (BD Biosciences, San Jose, CA, USA), using a combination of antibodies provided by BD Biosciences: CD45-V500, CD56-APC, CD16-V450, CD10-PECy7 and APC-H7, CD19-PerCP-Cy5.5, CD81-FITC, CD38-PECy7, CD138-PE, CD71-FITC, CD117-APC and adding CD117-BrilliantViolet 421 (BioLegend, San Diego, CA, USA). Briefly, 5 µl of each antibody was mixed to 100 µl of fresh BM sample and incubated for 15 minutes. The sample was then lysed and washed before acquisition. A median of 75 000 events were acquired and no less than 5 events were used to define a cell population. We decided to exclude CD34+ cells from our subset populations, because of the disease’s context (lymphoproliferative diseases) and the fact that CD34+ cells amounts are also related to patient gender and age (17), as well as to circadian cycles (21–23). Alongside FC analyses of cell populations, a BM differential blood count *via* both cytological analysis (data not shown) and Sysmex XN-1000™ Hematology Analyzer (Sysmex America Inc., IL, USA) (shown in **Table 2**) was performed.

**Table 2:** BM cell populations evaluation. Assessment of the presence of bone marrow cell populations by an automated analyzer.

Type of Cells	Median (range)	Median Percentage (range)
WBCs^1^	9940 (80-68420)	na^*^
Neutrophils	na^*^	59.1 (23.6-89.6)
Monocytes	na^*^	26.09 (0-79)
Lymphocytes	na^*^	9.2 (1.8-37.6)
NRBCs^2^	na^*^	13.1 (0-44.1)
IG^3^	na^*^	15.85 (0-44.1)

^1^White Blood Cells (WBCs) expressed in cell/µl; ^2^Nucleated Red Blood Cells (NRBCs); ^3^Immature granulocytes (IG); ^*^Not applicable (na)/not evaluated.

**Figure 1:**
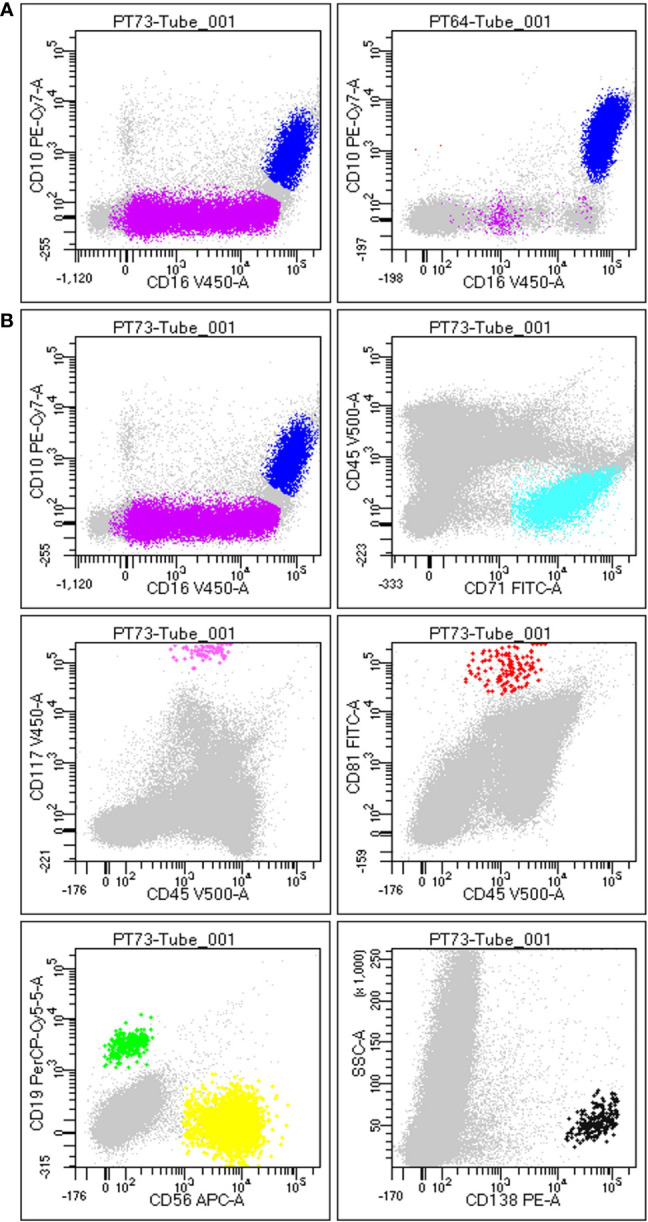
Gating strategy to define BM population. **(A)** Difference between a non-hemodiluted sample (left) and a hemodiluted sample (right), as shown by the presence of both immature (purple) and mature granulocytes (blue): the maturation line of granulocytes (including promyelocytes, myelocytes and metamyelocytes) is highlithed in purple and is highly present in nonHD samples (on the left), wherease it is almost absent in HD samples (on the right). **(B)** Each plot is the result of the different gating strategies used to define different populations, according to FSC/SSC and the specific markers. In details, plot 1 displays immature (SSC++/FSC++; CD45dim/+/CD16low/CD10neg) and mature granulocytes (SSC++/FSC++; CD45dim/+/CD10+/CD16+), plot 2 shows erythroblasts (SSClow/FSClow; CD45neg/CD71+), plot 3 displays mast cells (SSC++/FSC++; CD45dim/CD117hi), plot 4 exhibits hematogones (SSClow; CD45dim/CD10+/CD81hi), plot 5 shows B-cells (SSClow; CD45hi/CD19+/CD56neg) and T-cells (NK-like T-cells/NK; SSClow; CD45hi/CD19neg/CD56hi), and plot 6 displays plasma cells (SSClow/FSC+; CD138hi).

The antibody combinations employed to define each cell type is described in **Table 3**, and the result of gating strategy is shown in **Figure 1**.

**Table 3:** Flow cytometry definition of BM cell populations. Distribution of the main BM cell populations, according to their immunophenotype.

Type of Cells	Immunophenotype^1^	Median (range)
Immature granulocytes	SSC^++^/FSC^++^; CD45^dym/+^/CD16^low^/CD10^neg^	22.85% (0.4-57.2)
Mature granulocytes	SSC^++^/FSC^++^; CD45^dym^/CD16^++^/CD10^++^	12.6% (0.2-57.8)
IG/N ratio	na^2^	1.26 (0.02-5.2)
Mast cells	SSC^++^/FSC^++^; CD117^hi^/CD45^dym^	0.006% (0-0.11)
Plasma cells	SSC^low^/FSC^+^; CD138^hi^	0.2% (0-3)
Hematogones	SSC^low^; CD81^hi^/CD10^+^/CD45^dym^	0.9% (0-9)
B lymphocytes	SSC^low^; CD45hi/CD19^+^/CD56^neg^	1.6% (0-10)
NK-like T cells/NK	SSC^low^; CD45hi/CD19^neg^/CD56^hi^	0.4% (0.3-16)
Erythroblasts	SSC^low^/FSC^low^; CD45^neg^/CD71^+^	5.3% (0.4-12.4)

^1^According to the EuroFlow consortium; ^2^Not applicable (na).

### IG/N ratio definition

The immature granulocytes (IG)/mature (neutrophils, N) ratio is a mathematical relationship between IG and N aimed at simplifying the hemodilution evaluation of BM samples. Considering that Sorigue et al. and Julie Pont herself had already studied both the correlation between Pont’s IG/N and the formula defined by Holdrinet et al. (HI) (18, 20), defining an *R* of 0.8 (p-value <0.001) in healthy individuals, we decided to use IG/N ratio as reference for our considerations. We excluded the possibility to employ the HI as a reference, even though it is considered as the gold standard to assess hemodilution by most authors, since it requires both a parallel PB sampling and a parallel characterization and count of leukocytes and erythrocytes. Conversely, the IG/N ratio is straightforward and, particularly in the lymphoproliferative disease setting, is not biased by the pathology considered and/or by MRD-positive results. Thus, according to Pont et al. a cut-off IG/N ratio of 1.2 was employed to distinguish hemodiluted (<1.2) from non-hemodiluted samples (≥1.2).

### Minimal residual disease assessment

For all patients MRD measurement was performed by NGS to investigate the IgH/TCR rearrangement(s). Analyses have been conducted *via* LymphoTrack^®^ Dx IgH (FR1/FR2/FR3)/IgK/TCR assays on MiSeq™ System (Illumina Inc, San Diego, CA, USA) on DNA extracted from BM samples. MRD measurements have been quantified at a sensitivity of at least 10^-5^, using a LymphoQuant B-cell Internal Control. Data analysis was performed by the LymphoTrack^®^ MRD Software (*In vivo*scribe Inc, San Diego, CA, USA) (24). Undetectable MRD was assessed with at least 90% of confidence.

In ALL patients, MRD was performed also by FC, in parallel with NGS, employing FACSCanto™ II (BD Biosciences, San Jose, CA, USA). Quality control of the instrument was daily performed using FACSDiva™ CST IVD beads (BD Biosciences, San Jose, CA, USA). The panel used to measure MRD comprised CD45, CD19, CD20, CD10, CD58, CD123, CD34, CD22 for lineage B, and CD3, CD5, CD7, CD2, CD4, CD8, CD1a and TCRγ for the T-lymphoid compartment. The FC overall sensitivity was at 10^-4^, and MRD negativity was set under the 0.01%, according to standardized guidelines (25–27).

### Statistical and bioinformatic analyses

All bioinformatics and biostatistics analyses were conducted using personalized scripts and R packages. Pearson and Kruskal-Wallis tests were employed to evaluate correlations between cell populations included in the study, as well as parameters related to the patient’s cohort, such as age, gender and therapy regimens at the time of the BM aspirate sampling. Medians were used to define cut-offs for flow cytometry analysis of cellular sub-sets. The confidence interval considered was 95%. All results obtained shown a p-value of at least 0.001.

## Results

This was a multi-step study, initially focused on the comprehensive comparison of various FC-based approaches to assess BM hemodilution (according to the comparative analyses of different cell populations previously employed by several authors to this purpose), and then aimed at the development of a novel, original algorithm (named *ALLgorithMM*) for the hemodilution assessment in patients’ BM samples dedicated to MRD evaluations.

### Definition of cell types within BM aspirates

For each BM sample, cell populations with a well-known distribution in both PB and BM (i.e., plasma cells, mast cells, lymphocytes, erythroblasts and granulocytes) were characterized and quantified by FC, as described in Materials and Methods section. As shown in **Table 3** (for gating strategy refer to **Figure 1**), relevant sub-sets of marrow cells were defined by a specific immunophenotype and quantified as absolute percentage, according to the major EuroFlow consortium operating procedures (28). For immature granulocytes all the maturation stages are intended, including promyelocytes, myelocytes and metamyelocytes.

As explained in **Table 2** (see Materials and Methods), BM aspirates have been also analyzed *via* an automated counter to obtain the differential blood count together with the percentage of each cell population.

### Correlation between major populations present in BM samples

Several approaches to define hemodilution have been reported in the literature over time, mostly based on flow cytometric analysis and particularly in the context of acute leukemias (either ALL or Acute Myeloid Leukemia) (16–19, 29). These studies were taken as a starting point to define the most suitable approach to be applied to lymphoproliferative disease during post-treatment follow-up. Using the bioinformatic and statistical strategy described in section Materials and Methods (paragraph 2.5), all populations present in BM aspirates – previously employed for hemodilution assessment – were correlated, considering as reference the immature (IG)/mature granulocytes (neutrophils, N) ratio defined by Pont in 2018 (18), to highlight which one best contributes to the definition of BM hemodilution.

In the initial cohort of patients, IG/N ratio was <0.5 in 33/138 (24%) cases, between 0.5 and 1.2 in 36/138 (26%) samples and ≥1.2 in the remaining 69/138 (50%) cases, without distinction among the different diseases.

**Figure 2:**
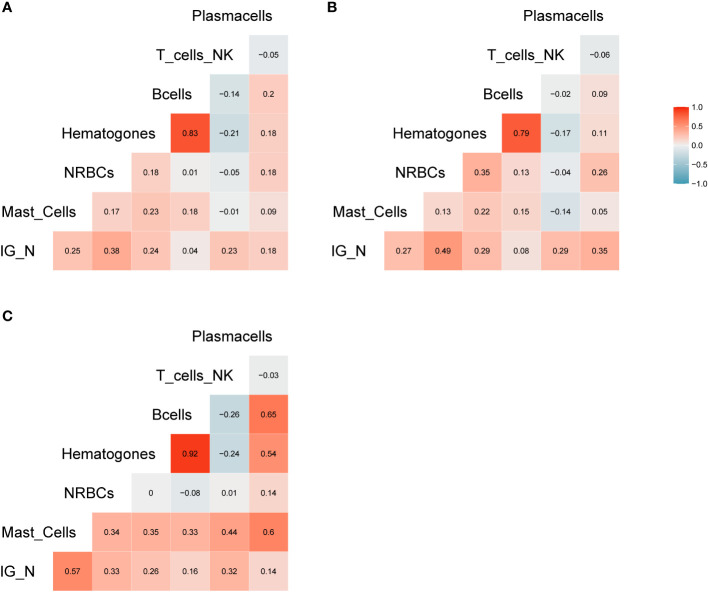
Correlation analysis. **(A)** Correlation matrix related to all samples included in the study; the relative matrices for MM **(B)** and ALL patients **(C)**. Positive correlations are displayed in red, while negative correlations are displayed in blue. Color intensity is defined by the correlation coefficient. Overall, the Pearson’s rho (*R*) for IG/N vs. mast cells and erythroblasts was ~0.3, with p<0.0006.

The amount of different cell populations (as described above and listed in **Table 3**), such as B-lymphocytes precursors and erythroblasts, was correlated to the IG/N ratio. Results are shown in **Figure 2**, highlighting a mild but highly significant correlation with mast cells and erythroblasts (*R*=0.25 and *R*=0.38, p-value = 0.0006 and 0.00000526, respectively), regardless of the hematological disease studied; on the contrary, no correlations were found with B- nor with T-cells. Hematogones showed a lower correlation (*R*=0.24), as compared to the other cells’ population. However, due to the pathological context (mainly involving the lymphoid lineage) and to the immunomodulatory effects of therapies employed, we decided not to take hematogones into consideration for further development of the study.

### The construction of the *ALLgorithMM*

**Figure 3:**
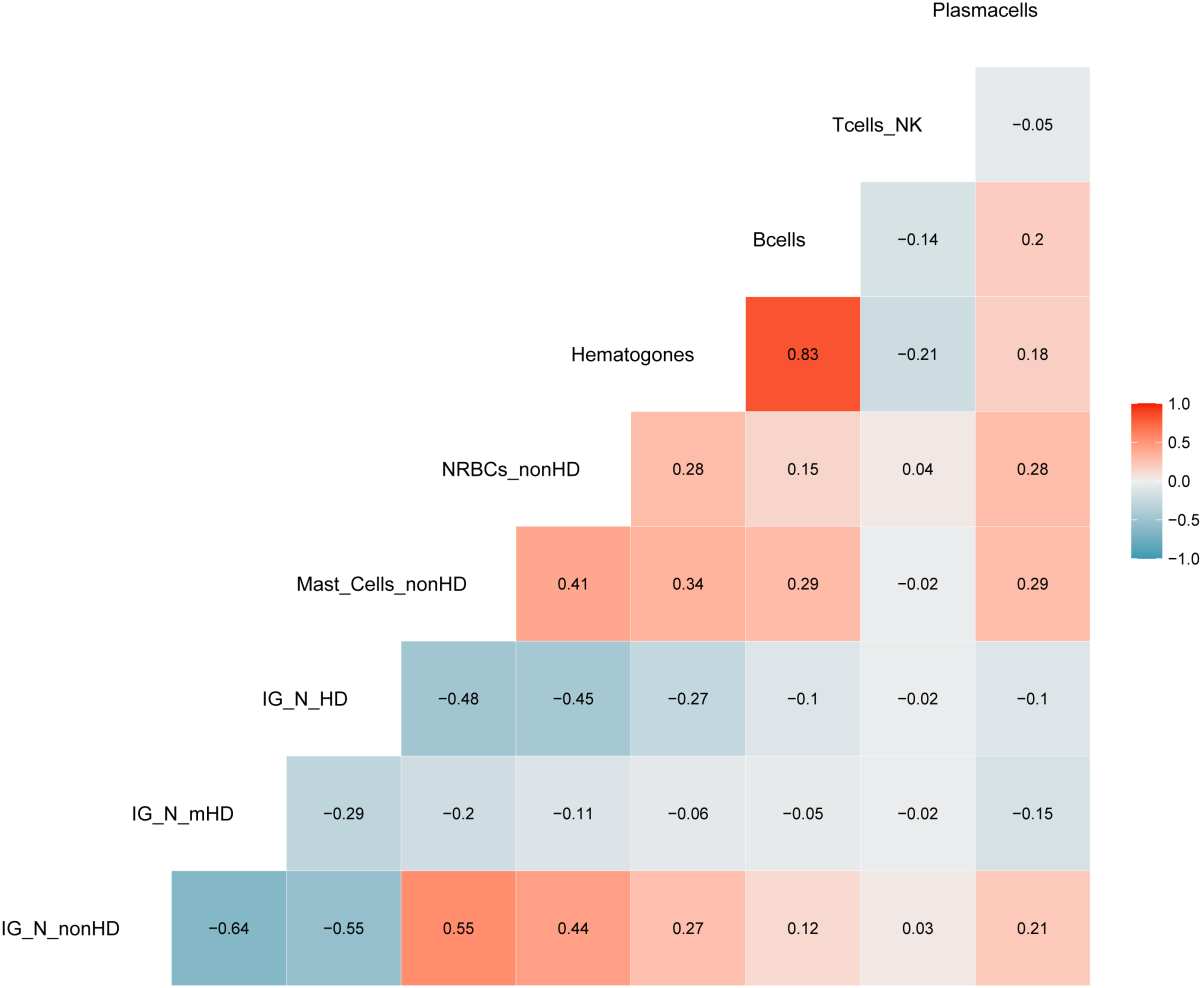
Correlation analysis with categorized parameters. Positive and negative correlations are presented in red and blue, respectively. Correlation coefficients are related to the color intensity. Once defined the cut-offs for the three parameters (IG/N, mast cells and erythroblasts (nucleated red blood cells, NRBCs), a higher correlation was observed between these categorized variables as compared to the same continuous variables shown in **Figure 2**.

The aforementioned correlations highlighted an average but highly significant correlation between IG/N ratio, erythroblasts and mast cells; the absolute percentages of cell populations can be variable in BM regardless of hemodilution, and must be taken into consideration. Thus, we integrated these three parameters to develop a simple (one tube-based), reliable and reproducible algorithm able to define the degree of BM hemodilution, named ALLgorithMM. This integration was also rupported by the categorization of the reference variables, that highlighted a stronger correlation (R>0.44; p<9.14e10^-8^), and confirmed we were able to correctly define BM samples hemodilution (**Figure 3**).

**Figure 4:**
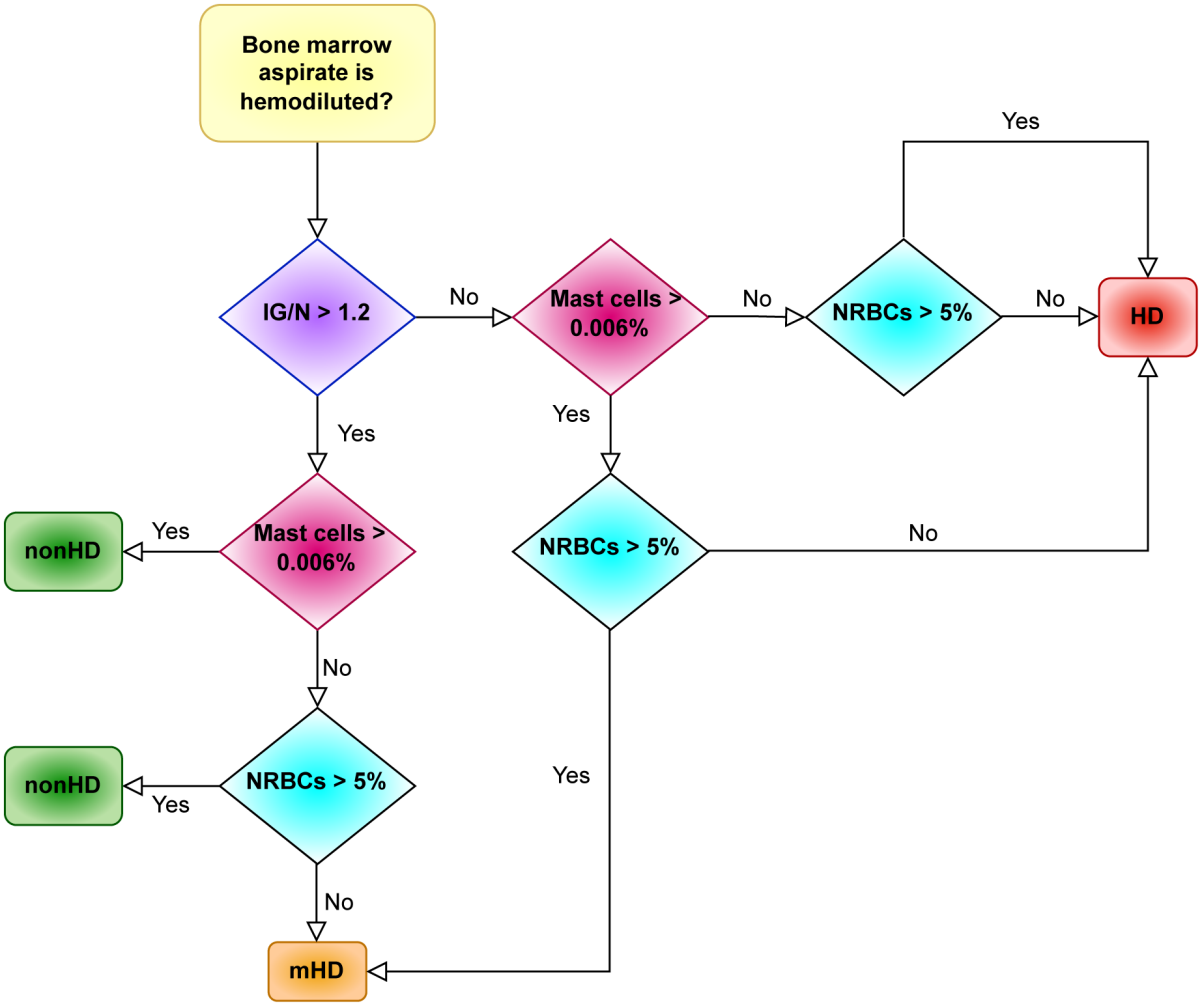
The *ALLgorithMM* sequence. For each BM aspirate, the definition of either hemodiluted or non-hemodiluted sample is the result of at least 2 out of 3 parameters, as analyzed by flow cytometry in the BM sample. Immature granulocytes (IG); mature granulocytes/neutrophils (N); erythroblasts (nucleated red blood cells, NRBCs); hemodiluted (HD); mildly hemodiluted (mHD); non-hemodiluted (nonHD).

Starting from the IG/N ratio and factoring for the percentages of erythroblasts and mast cells, ALLgorithMM allows to define three types of BM samples: hemodiluted (HD), mildly hemodiluted (mHD) and non-hemodiluted (nonHD), as highlighted in red, orange and green, respectively, in **Figure 4**. The cut-offs used in the ALLgorithMM derived from the previously described Pont IG/N ratio, also confirmed in our dataset, whereas the cut-offs for mast cells and erythroblasts where obtained from the median of our collected data. Notably, a biomarker-based cut-off was chosen, instead of and outcome-based one, since no association with outcome could be defined due to the short median patients’ follow-up and the lack of survival events.

As resumed in **Figure 4**, this algorithm sequentially evaluates the IG/N ratio, the mast cells and the erythroblasts: the decisional ALLgorithMM proceeds by stratifying the attained results according to pre-defined cut-offs (i.e., 1.2 for IG/N ratio, 0.006% for mast cells and 5% for erythroblasts/NRBCs), providing the final definition of samples quality. To efficiently analyse the selected parameters, we developed a 1-tube 5-colors FC panel, easily managable and simple to analyse, including CD10-APCH7, CD16-V450, CD45-V500, CD117-APC and CD71-FITC (antibodies provided by BD Biosciences, San Jose, CA, USA). Notably, the use of only 5 markers leaves room to analyse additional, pathology-related markers, according to the need to evaluate the presence of residual cells (e.g., in MM, CD38 and CD138 could be added and used to define and to verify the presence of plasma cells).

Finally, we observed that in most cases (220/233, 94.4%), the combined employment of IG/N, mast cells and erythroblasts allowed to confirm the data derived just from the IG/N ratio evaluation. In the remaining cases (13/233, 5.6%), IG/N ratio did not adequately describe BM hemodilution; in fact, in these samples, the presence of just metamyelocytes, in absence of the other elements of granulocyte maturation line, misled the “IG” count, thus resulting in a wrongly defined not-hemodiluted result. Therefore, the combination of different parameters contributed to an improved definition of BM hemodilution, which might be critical, particularly in mildly hemodiluted samples.

### IG/N, mast cells and erythroblasts define 3 well-distributed groups

Once defined, the *ALLgorithMM* was prospectively validated on samples consecutively collected from 42 MM and 27 ALL patients (57 and 38 samples, respectively), whose MRD was measured either by NGS or by FC, in the context of daily clinical practice. Overall, 12/57 MM and 10/38 ALL samples resulted HD, and 18/57 MM and 5/38 ALL were instead mHD.

**Figure 5:**
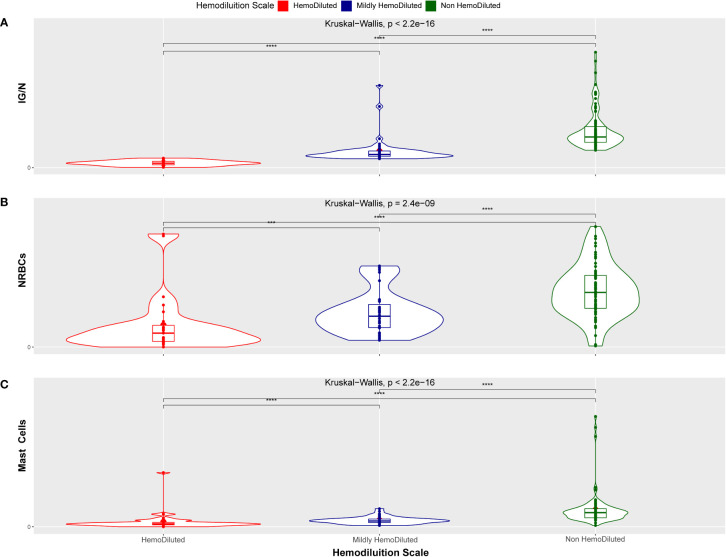
Kruskal-Wallis distribution. Hemodiluted (HD, red), mildly hemodiluted (mHD, blue) and non-hemodiluted (non-HD, green) sample distribution related to the IG/N ratio **(A)**, to the erythroblasts (nucleated red blood cells, NRBCs) **(B)** and to the mast cells presence **(C)**. The distribution between groups has a p-value inferior to 2.4e10-9, indicating a statistically highly significant tendency. (***p < 0.0005 and ****p < 0.00005).

A Kruskal-Wallis test was used to confirm the ability of the *ALLgorithMM* to define significantly homogeneous clusters of samples, defined as HD, as nonHD or mHD. The results shown in **Figure 5** enlighten how the three parameters included in the *ALLgorithMM* (a - IG/N; b – erythroblasts; c- mast cells, as in the figure) have been able to significantly stratify patients in distinct groups (i.e., HD, mHD and nonHD) (p-value<2.4e10^-9^). The other investigated cell populations did not show this behavior (data not shown) and, therefore they were not taken into consideration for the *ALLgorithMM*.

**Figure 6:**
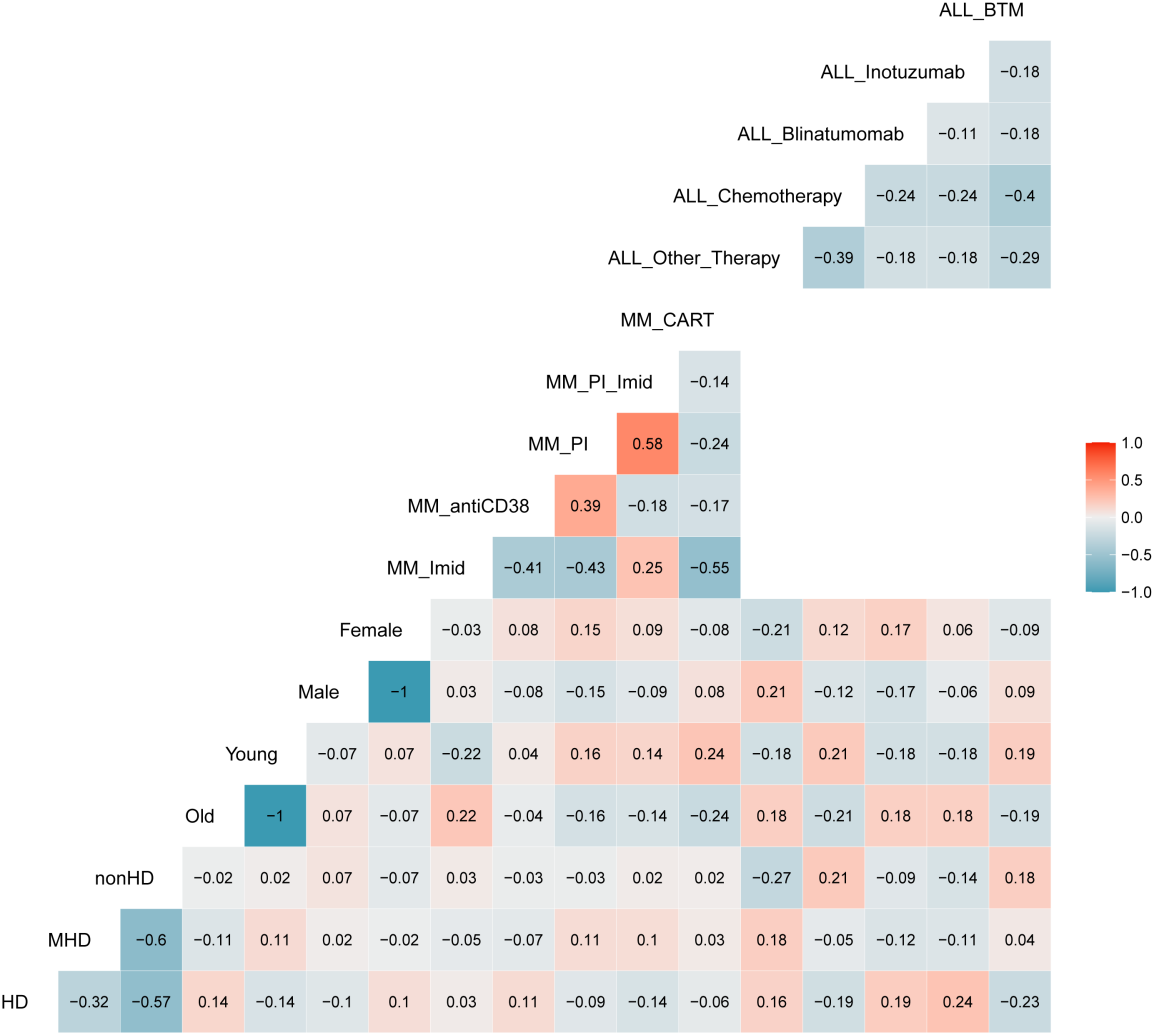
Correlation matrix between *ALLgorithMM*-defined BM hemodiluted (HD), mildlyHD (mHD) and non-hemodiluted (nonHD) vs. age (old or young), gender (female or male) and therapy used at the time of the sampling. Blue and red stand for negative or positive correlations, respectively. Color intensity is proportional to the correlation coefficients. The figure shows an absence of correlation between the 3 HD groups and others variables, confirming the robustness of the *ALLgorithMM*.

We finally investigated whether clinical variables (such as the therapy provided at the time of BM sampling, patients’ age or gender) might influence the hemodilution evaluation by *ALLgorithMM*. As shown in **Figure 6**, there was no significant correlation between hemodilution definition and the variables aforementioned, supporting the robustness of the validated *ALLgorithMM* approach.

### The impact of hemodilution on MRD measurement

**Figure 7:**
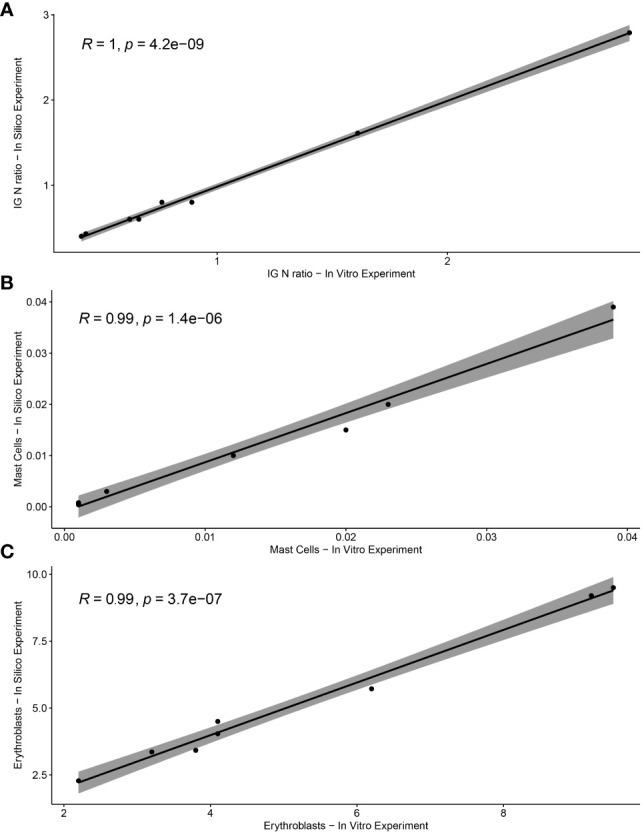
Correlation between *in vitro* and *in silico*-calculated data of *ALLgorithMM* parameters. The Pearson’s rho (R) accompanied by the relative p-value for **(A)** IG/N ratio, **(B)** mast cells, and **(C)** erythroblasts.

To validate the *ALLgorithMM*, we performed *in vitro* serial dilutions of BM in PB derived from two MM patients, aiming also at measuring MRD according to the diverse hemodilution levels. Starting from the non-hemodiluted BM sample, we simulated 4 different scenarios: a) sample as-it-is, after BM collection (nonHD), b) sample diluted with PB, to obtain an MHD sample, c) sample diluted with PB to attain a fully hemodiluted sample (HD), d) sample diluted with PB, to obtain a nearly HD-MHD sample (MHDlow). The 4 samples *per* patient were then analyzed by using the *ALLgorithMM*, to confirm the hemodilution status, as shown in **Table 4**, including the relative *in silico*-derived measures. Briefly, the initial BM sample was diluted with patient-derived PB to get the above-mentioned four scenarios, still maintaining the original BM WBC count for each sample. *In silico* and *in vitro* data were then tested to assess the results’ linearity, obtaining a Pearson correlation coefficient of 1 with p=4.2e^−09^ for IG/N ratio, a *R*=0.99 with p=1.4e^−06^ for mast cells and *R*=0.99 with p=3.7e^−07^ for erythroblasts, as shown in **Figure 7**.

**Table 4:** Changes in the composition of BM cell populations in serial hemodilution experiments. Distribution of parameters used in the *ALLgorithMM* to define hemodilution of BM samples, within 4 different scenarios simulated for each patient: nonHD (sample as it is), MHD (sample diluted with PB to obtain an MHD sample), MHDlow (sample diluted with PB to obtain an MHD sample nearly HD), and HD (full hemodiluted sample). In parentheses are represented *in silico*-derived values, as expected by applying serial dilutions of samples to simulate the 4 different cases.

Type of Cells	^1^pt1 nonHD	pt1 MHD	pt1 MHDlow	pt1 HD	pt2 nonHD	pt2 MHD	pt2 MHDlow	pt2 HD
IG/N ratio	2.79	0.89 (0.8)	0.66 (0.6)	0.43 (0.4)	1.61	0.76 (0.8)	0.62 (0.6)	0.41 (0.4)
Mast cells	0.003%	0.001% (0.0008%)	0.001% (0.0006%)	0.001% (0.0004%)	0.039%	0.023% (0.02%)	0.02% (0.015%)	0.012% (0.01%)
Erythroblasts	9.5%	6.2% (5.72%)	4.1% (4.04%)	3.2% (3.36%)	9.2%	4.1% (4.5%)	3.8% (3.42%)	2.2% (2.28%)

^1^pt: patient.

MRD was then analyzed by NGS in the same samples and results are shown in **Table 5**: as expected, the artificial impairment of BM aspirate quality caused an overall underestimation of residual disease measurements. In fact, in patient 1 (pt1), whose residual disease was measured in the order of 10^-5^ cells in nonHD sample, MRD measurement was not reliable in both MHD cases, and also underestimated in the HD sample. On the contrary, in patient 2 (pt2) MRD was undetectable in nonHD sample, with a confidence of 97% at 10^-5^: this result was progressively less confident in hemodiluted samples, suggesting that low quality BM aspirates might impair also the reliability of MRD-negative results.

**Table 5:** MRD evaluation in hemodiluted BM samples. NGS-based MRD measurements for two MM patients (pt) whose BM samples were progressively diluted with PB, to obtain 4 different cases: nonHD, MHD, MHDlow, and HD. MRD measures are quantified by the LymphoTrack^®^ MRD Software (*In vivo*scribe Inc, San Diego, CA, USA). In bold: Results are an estimate of the clonal frequency of the prevalent rearrangement(s) detected at diagnosis. For each sensitivity level indicated, the statistical confidence of the result is reported.

	^1^pt1 nonHD	pt1 MHD	pt1 MHDlow	pt1 HD	pt2non HD	pt2 MHD	pt2 MHDlow	pt2 HD
**MRD measure**	1.74x10^-5^	2.44x10^-5^	1.46x10^-5^	9.11x10^-6^	0	0	0	0
**Confidence 10^-3^**	100%	100%	100%	100%	99.99%	99.99%	99.99%	99.99%
**Confidence 10^-4^**	99.74%	99.99%	99.99%	99.99%	99.99%	99.99%	99.99%	84.71%
**Confidence 10^-5^**	80.37%	10.75%	15.12%	25.86%	96.68%	77.01%	80.51%	14.51%
**Confidence 10^-6^**	0.18%	1.52%	0.06%	0.11%	12.45%	7.57%	3.71%	1.52%
**Result**	**^2^POS(80% at 10^-5^)**	**^3^NEG(99% at 10^-4^)**	**NEG(99% at 10^-4^)**	**NEG(99% at 10^-4^)**	**NEG(97% at 10^-5^)**	**NEG(99% at 10^-4^)**	**NEG(99% at 10^-4^)**	**NEG(99% at 10^-3^)**

^1^pt: patient; ^2^POS (positive); ^3^NEG (negative).

According to the *ALLgorithMM*, a range of hemodilution grades could be highlighted, with high percentage of HD and/or mHD samples (22% and 25%, respectively) among all evaluated patients, as shown in **Table 6**, section a. These BM samples were also addressed to MRD measurement. MRD was assessed by FC (for ALL patients only) and by NGS for MM and ALL Ph-negative patients, as described in the Materials and Methods section.

**Table 6:** a) Hemodilution assessment through the *ALLgorithMM*. Samples are stratified into three main groups, defined as hemodiluted (HD), mildly hemodiluted (mHD) and non-hemodiluted (nonHD) by the result of the *ALLgorithMM* application. b) The impact of hemodilution on MRD evaluation. For each category, the number and the relative percentage are referred to hemodiluted or mildly hemodiluted samples, enlightening the potential impact of hemodilution on the minimal residual disease (MRD) measurement results, as assessed by flow cytometry (FC) or by molecular (mol, *via* NGS) approaches.

a)		HD	mHD		nonHD
** ALL**		17/69 (25%)	8/69 (12%)		44/69 (63%)
** MM**		33/150 (22%)	46/150 (31%)		71/150 (47%)
CAR-T*		2/14 (14%)	4/14 (29%)		8/14 (57%)
** Tot**		52/233 (22%)	58/233 (25%)		123/233 (53%)
**b)**	**FC MRD neg**	**FC MRD pos**	**mol MRD neg**	**mol MRD pos**	**mol MRD pnq**
** ALL**	25 (8; 32%)	23 (9; 39%)	26 (6; 23%)	13 (7; 54%)	7 (2; 29%)
** MM**	na**	na	38 (27; 71%)	16 (4; 25%)	32 (18; 56%)
** Tot**	25 (8; 32%)	23 (9; 39%)	64 (33; 52%)	29 (11; 38%)	39 (20; 51%)

*CAR-T is referred only to MM patients underwent to CAR-T therapy in general (i.e., anti-BCMA CAR-T) after at least 56 days from the infusion; **Not applicable (na), as our center does not perform MRD evaluations by flow cytometry for MM patients.

In general, FC MRD measure resulted undetectable in 25/48 (52%) cases and positive in 23/48 (48%) ALL samples. Molecular MRD measurements were negative in 26/46 (57%), positive in 13/46 (28%) cases and positive non-quantifiable (PNQ) in 7/46 (15%) ALL BM aspirate samples. In MM patients, molecular MRD was undetectable in 38/86 (44%), PNQ in 32/86 (37%), and positive in 16/86 (19%) cases.

Of the 26 ALL samples tested negative for molecular MRD, 6 (23%) were hemodiluted and/or mildly hemodiluted. Of the ALL samples tested by FC, 8/25 (32%) negative and 9/23 (39%) positive cases were either mildly or highly hemodiluted. In MM, undetectable MRD results were reported in 27/38 hemodiluted cases (71%).

All MRD results are summarized in **Table 6**, section b. For CAR-T MM patients, MRD was not provided for this study.

Overall, 52% of MRD-negative cases evaluated with NGS were HD or mHD (17/64, 27% and 16/64, 25%, respectively), whereas 48% were nonHD. For MM, only 29% (11/38) of BM used to measure MRD passed the hemodilution quality control assessment, whereas 34% (13/38) of cases were highly hemodiluted and 37% (14/38) were mildly HD. ALL samples were instead HD in the 11% of cases (3/26), mHD in 3 cases over 26 (11%) of evaluation and the majority (77%, 20/26) were non-hemodiluted.

## Discussion

Over the last years, the advancement of molecular and flow cytometry methods to assess MRD in lymphoproliferative disorders has led to a game-changer scenario, where it has become fundamental to obtain well-prepared samples to provide a comprehensive and representative snapshot of the BM tumor landscape and distribution. Nevertheless, several non-standardized protocols, mainly based on flow cytometry, have been investigated to evaluate the quality of bone marrow sampling, without anyhow leading to a well-defined and reliable method, easily applicable in lymphoproliferative disease regardless of therapy, age and/or gender of the patient.

Here, we set-up and validated a novel approach to assess BM hemodilution in lymphoproliferative disorders, named *ALLgorithMM*, based on the measurement of three parameters (immature (IG)/mature granulocytes (neutrophils, N) ratio, mast cells and erythroblasts amount), which can easily and objectively stratify BM samples according to the extent of hemodilution.

The *ALLgorithMM* has been validated on BM samples of patients after therapy, excluding BM with active disease (defined as > 5% blast cells and >5% of plasma cells for ALL and MM samples, respectively).

The combination of the three selected parameters derived from the observed mild, but highly significant correlation between IG/N ratio, nucleated red blood cells and mastocytes, which prompted the combined employment of these parameters to evaluate BM hemodilution; on the contrary, neither lymphocytes nor hematogones (both commonly employed to evaluate hemodilution) seemed informative enough to this purpose. The peculiar choice of these parameters to assess hemodilution was supported by several observations. First of all, the lymphoproliferative hematological diseases are characterized by an alteration in the number of cells of the lymphoid lineage. Secondly, in this clinical context, the immunomodulatory/lymphodepletive role of treatments might cause alterations and/or changes in the composition of the BM niche as well as of the microenvironment. Moreover, either a decrease of B-cell precursors or the expansion of B and T cells can occur after stem cell transplant and/or CAR-T infusion (19, 30). Thirdly, the typical absence of mast cells and of erythroblasts in PB supports their role as references in this approach.

All the above-mentioned reasons strongly supported the choice to focus on the integration of information deriving from mast cells, erythroblasts and IG/N ratio, confirming the originality and the reliability of *ALLgorithMM*, particularly in the context of lymphoproliferative diseases.

In addition, by using three different parameters, *ALLgorithMM* might be potentially applied also to other hematological disorders, without incurring in biased data, possibly caused by the over-representation of cells’ population directly involved in the disease. Finally, the *ALLgorithMM* prevents all issues related to the intra- and inter-patient intrinsic differences in the BM distribution of cells populations thanks to the combined employment of three parameters, i.e., the IG/N ratio and two cell populations (mastocytes and erythroblasts) percentages.

As a major added value, *ALLgorithMM* refines the results obtained just by the IG/N ratio, particularly when the immature granulocytes configuration is hard to be defined, for instance when the granulocyte maturation and/or composition might be compromised or when the granulopoiesis is just represented by the presence of metamyelocytes. In the present study, the hemodilution assessment has been re-adjusted with respect to the evaluation performed by the IG/N ratio in 5.6% of cases (13/233), thanks to the inclusion of both mastocytes and erythroblasts evaluations. Of these, 5/13 (38%) were defined hemodiluted thanks to mast cells and erythroblasts frequencies clearly under the cut-offs defined in the *ALLgorithMM* (0.006% and 5%, respectively). Similarly, the other 8/13 samples (62%) defined non-hemodiluted just according to IG/N ratio, were re-defined mildly hemodiluted thanks to the use of mast cells and erythroblasts; in fact, in these samples, the presence of metamyelocytes as the only granulocytic cell lineage-representing cells, caused and incorrect assessment of the immature granulocytes count, thus suggesting the importance of additional parameters to correctly define such borderline situations.

Furthermore, the *in vitro* simulation of BM hemodilution scenarios, described in paragraph 3.5, allowed both to validate the *ALLgorithMM* and to highlight the impact of hemodilution on MRD assessment. In fact, the BM serial dilution with PB, by simulating either mildly or fully hemodiluted situations, caused an overall underestimation of residual disease measurements, with a progressively decreased MRD values and/or NGS results’ confidence in low quality BM samples.

The need to perform good-quality BM sample aspirates, highly representative of the tumor cells distribution, both not diluted and not contaminated by PB cells, is increasingly higher in hematologic diseases, mainly due to the growing role gained by MRD measurements after therapy for prognostication and for treatment tailoring. The high frequency of MRD-negative measurements observed in this study in both mildly and fully hemodiluted samples indicates a possible recurrent underestimation of MRD measurements, which overall might impair the correct assessment of the depth of response to therapy and therefore possibly cause wrongly supported prognostications and/or clinical decisions. This highlights the importance to include a reliable evaluation of BM hemodilution in the daily practice as an important quality control step, allowing to point out low quality BM samples and to report them to clinicians, to possibly plan a new BM evaluation, if needed.

In this context, the *ALLgorithMM* proved to be reproducible and easily applicable to evaluate hemodilution in BM samples of patients affected by lymphoproliferative diseases, such as ALL and MM. The routine application of this method can support a correct assessment of MRD, reducing the possibility of false negative results, providing essential samples quality information. Moreover, the *ALLgorithMM* can be employed also for samples collected both at diagnosis and at relapse (i.e., in highly infiltrated samples), to correctly assess the disease burden at that specific time-point, since it might be underestimated, due to bad-quality sampling. This becomes especially relevantwhen the effective amount of tumor burden is diriment for patients’ inclusion in protocols and/or in clinical trials, or is crucial for clonotype assessment by NGS and in the identification of the disease grade.

## Data availability statement

The raw data supporting the conclusions of this article will be made available by the authors, without undue reservation.

## Ethics statement

Ethical review and approval were not required for the study on human participants in accordance with the local legislation and institutional requirements. The patients/participants provided their written informed consent to participate in this study.

## Author contributions

Conception: IV. Experimental analyses: IV, SA, MB, BT, and IP. Manuscript preparation: IV, EB, and CT. Statistical analysis VS and IV. MM, AP, GM, AC, IR, LP, PT, CS, CP, KM, SR, EZ, MA, and MC provided patients and tissue materials. All authors contributed to the article and approved the submitted version.

## Funding

Ministero della Salute (RC-2022-2773359), Associazione Italiana Contro le Leucemie - Linfomi e Mieloma (AIL), Italian Association for Cancer Research (AIRC) [IG2018-22059].

## Acknowledgments

The authors would like to recognize Miriam Scotto di Fasano for the support in the experimental analysis and Stefano Delle Vedove for supporting the statistical analysis. We also would like to thank all the Study Coordinators and the Research Groups involved in the Seràgnoli Institute, Bologna, Italy.

## Conflict of interest

The authors declare that the research was conducted in the absence of any commercial or financial relationships that could be construed as a potential conflict of interest.

## Publisher’s note

All claims expressed in this article are solely those of the authors and do not necessarily represent those of their affiliated organizations, or those of the publisher, the editors and the reviewers. Any product that may be evaluated in this article, or claim that may be made by its manufacturer, is not guaranteed or endorsed by the publisher.
